# Use of Lumen-Apposing Stents for the Treatment of Postsurgical Fluid Collections: A Case Series and a Review of Literature

**DOI:** 10.1155/2019/7656950

**Published:** 2019-01-23

**Authors:** Priyanka Priyanka, William Hsueh, John Nasr

**Affiliations:** ^1^Department of Medicine, WV University Hospital, Morgantown, WV, USA; ^2^Department of Medicine, Division of Gastroenterology, WVU University Hospital, Morgantown, WV, USA

## Abstract

Lumen-apposing metal stents (LAMS) use in gastrointestinal endoscopy has been on the rise for various indications for the last few years. Currently, LAMS is a well-established treatment for post-pancreatitis peri-pancreatic fluid collections and walled-off necrosis (WON), but it is still not a standard of care in the treatment of post-surgical fluid collections (PSFC). Most of the earlier studies for treatment of PSFC utilized double pigtail plastic stents (DPS). We present a series of 3 cases where LAMS was successfully used for PSFC drainage. The cases include a patient with perigastric abscess after Whipple's procedure, a case of peri-pancreatic collection after distal pancreatectomy, and a patient with peri-pancreatic fluid collection after right partial hepatectomy and splenectomy due to lacerations from a motor vehicle accident.

## 1. Introduction

Post-surgical fluid collections (PSFCs) can be an important cause of morbidity and mortality depending upon the nature of the surgery and site of the collection. Over the last few decades, percutaneous radiologic drainage (PCD) has been considered the first-line treatment of PSFC with a success rate of 80-100% [1, 2]. Due to its lower morbidity, mortality, and costs, percutaneous drainage has become widely accepted over open surgical drainage.

With advances in therapeutic endoscopy over the past few years, there has been increasing use of plastic and metal stents as an attractive alternative to PCD, especially in the treatment of pancreatic and peripancreatic fluid collections and walled-off necrosis (WON) [3, 4]. Recently, the use of these stents has been extended for postsurgical fluid collection drainage [5–7]. Newer studies have contributed to the understanding that endoscopic ultrasound guided drainage (EUS-GD) and PCD are equally effective and safe in treatment of PSFCs [2]. In addition, the advantages of endoscopic drainage over PCD include higher clinical success rate, lower complication rate, less procedure related mortality, lack of an external drain, improvement in quality of life, lower total costs, and fewer complications related to a drain such as fluid losses and bleeding. Most of the earlier studies used double pigtail plastic stents (DPS) [6, 7]. Lumen-apposing metal stents (LAMS) use in gastrointestinal endoscopy has been on the rise for the past 2-3 years for various indications since its first clinical use in 2012. Recent studies have suggested the superiority of LAMS to DPS for WON drainage; however the scientific literature available with regard to PSFCs is scarce with only one study reporting their use for this indication so far [3, 5]. We present a series of cases of successful use of LAMS for drainage of PSFCs.

## 2. Case Series

### 2.1. Patient 1

**Figure 1:**
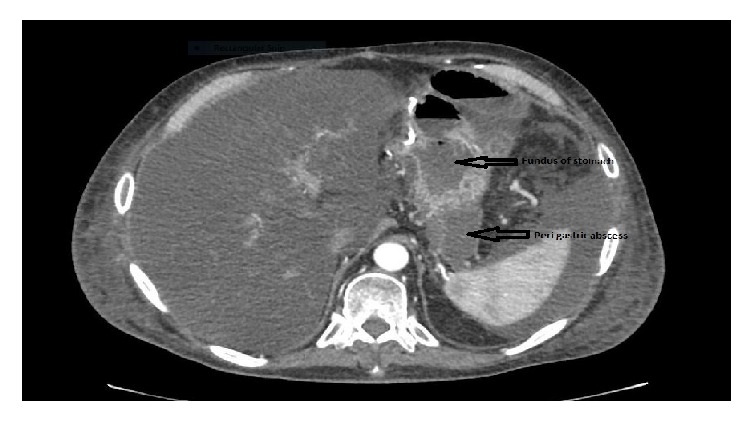
Patient 1, CT abdomen showing perigastric abscess.

**Figure 2:**
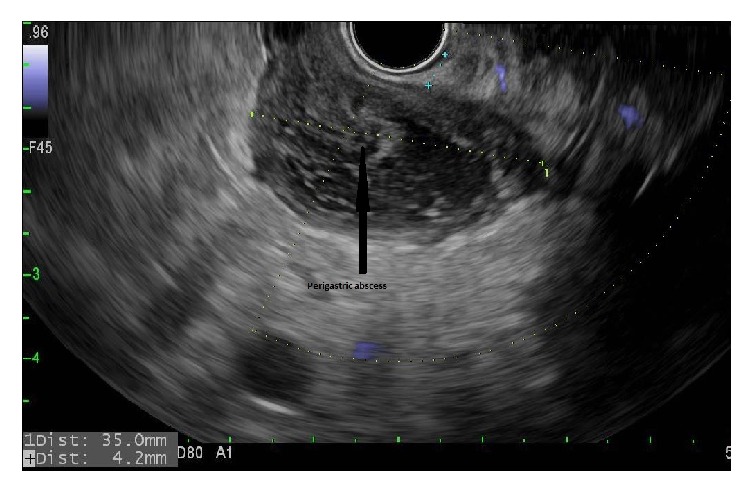
Patient 1, EUS confirming perigastric abscess in fundal area.

A 54-year-old male with a history of renal cell carcinoma, pancreatic adenocarcinoma stage II (T2 N1 3/5 lymph nodes positive), having previously received chemotherapy followed by stereotactic body radiation therapy (SBRT) presented 5 months after the Whipple's surgery with failure to thrive, fatigue, and nausea. Exam was unremarkable and laboratory investigations revealed albumin of 1.3 mg /dl, bilirubin of 2.8 mg/dl, mainly conjugated, serum alkaline phosphatase of 825 U/L, and CA 19-9 of 81.4 (normal <37 U/ml). Computed tomography scan (CT) of abdomen showed a perigastric abscess adjacent to the fundus (Figure 1). Endoscopic ultrasound (EUS) was suggestive of 35 mm anechoic, heterogeneous, well-circumscribed fluid collection in the immediate perigastric area surrounding the fundus (Figure 2). Under endosonographic, fluoroscopic, and Doppler guidance, a 10 x 10 mm LAMS was placed from the stomach into the fluid collection with drainage of pus.

**Figure 3:**
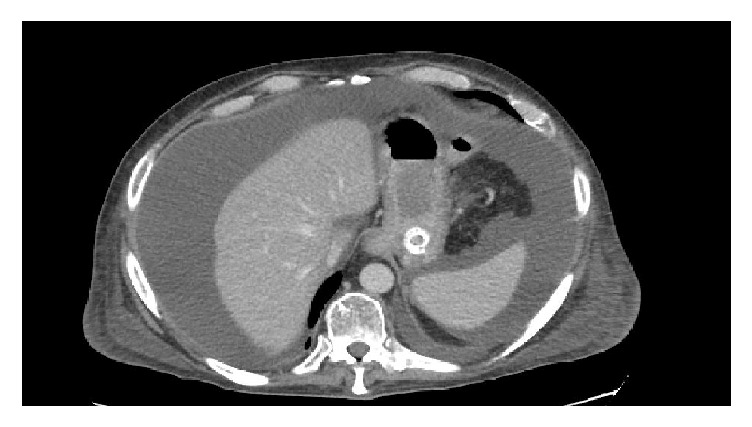
Patient 1, CT scan abdomen showing resolution of perigastric abscess.

The patient improved clinically along with significant improvement in his bilirubin and alkaline phosphatase after the procedure. Repeat CT abdomen after one week of stent placement showed a near-complete resolution of the abscess (Figure 3), although he had developed ascites by this time, likely due to presence of severe hypoalbuminemia. Removal of the stent was planned after 3 weeks of placement. However, the patient was readmitted 3 weeks later with respiratory failure and altered mental status. His family elected to provide supportive care only and he died shortly thereafter.

### 2.2. Case 2

**Figure 4:**
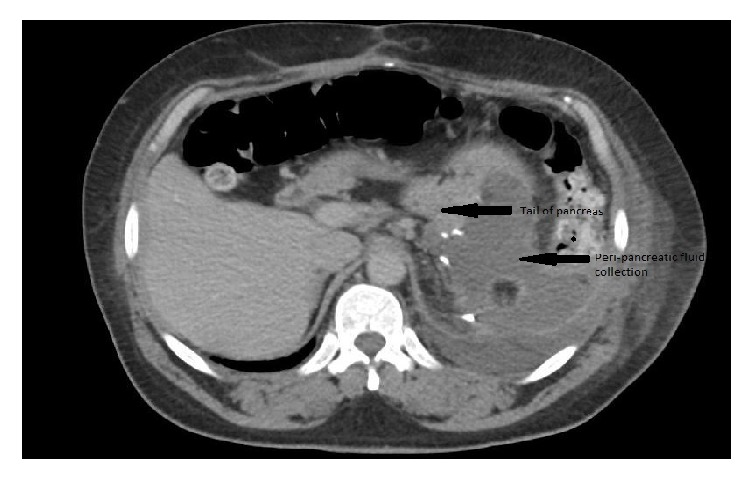
Patient 2, CT scan abdomen showing fluid collection around the tail of pancreas.

**Figure 5:**
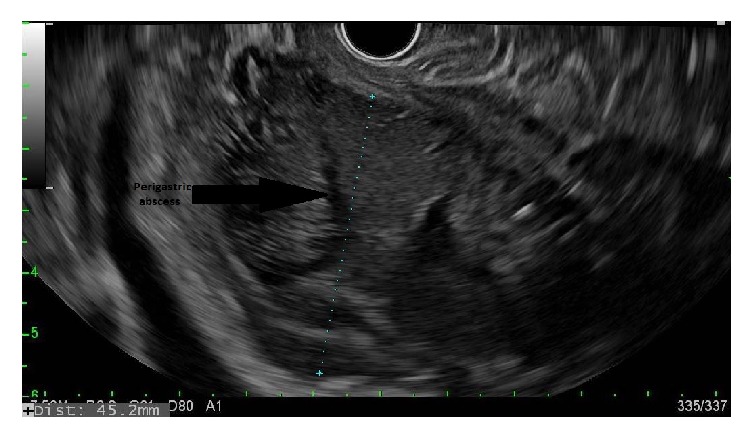
Patient 2, EUS confirming peripancreatic fluid collection around tail of pancreas.

**Figure 6:**
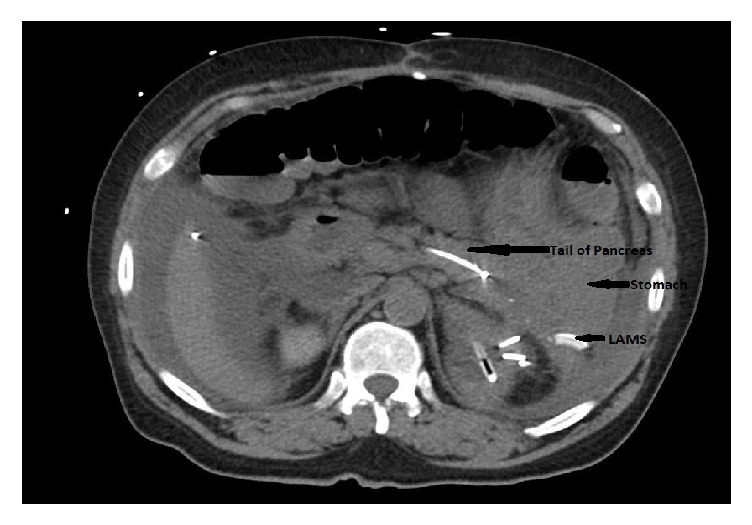
Patient 2, CT scan showing resolution of peripancreatic fluid collection.

54-year-old male underwent distal pancreatectomy with splenectomy for treatment of pancreatic neuroendocrine tumor. In the immediate postoperative period, patient developed a pancreatic fluid leak from the tail of pancreas and an intra-abdominal drain was placed that was removed after it stopped draining. CT scan showed interval increase in the size of the rim enhancing fluid collection around tail of pancreas 1 month after drain removal (Figure 4). ERCP was performed for suspected pancreatic duct (PD) leak and confirmed a leak from the tail of the pancreas. Endoscopic pancreatic sphincterotomy was done with placement of a 5 Fr x 13 cm pancreatic duct stent with internal barbs. Subsequently, EUS showed a well demarcated, hypoechoic, heterogeneous collection, adjacent to the tail of the pancreas about 6.5 cm in the largest dimension (Figure 5). Under endosonographic guidance, a 15 mm x 10 mm LAMS was placed from the stomach into the fluid collection with drainage of a large amount of pus. CT scan of abdomen after 1 month showed decrease in the size of the previously demonstrated LUQ rim-enhancing fluid collection (Figure 6). Unfortunately, patient later had a neurological event that led to his demise, prior to stent removal.

### 2.3. Case 3

A 34-year-old male presented to hospital after a motor vehicle accident. Patient was hypotensive on arrival and underwent exploratory laparotomy, splenectomy, embolization of hepatic vessels, and right-sided partial hepatectomy due to grade V liver laceration. Postoperatively, the patient developed bilious drainage from an intra-abdominal drain and underwent ERCP for suspected bile leak. ERCP revealed a leak from the right biliary system; therefore a 10 Fr x 5 cm plastic biliary stent was placed. A week later, the patient continued to have high output of amylase-rich fluid from a separate intra-abdominal drain which was suspicious for a pancreatic duct leak. Repeat ERCP with pancreatogram revealed a leak from the pancreatic tail for which a 5 Fr x 13 cm pancreatic duct stent was placed and the biliary stent was upsized to a 10 mm x 4 cm covered self-expanding metallic stent (SEMS) due to a persistent biliary leak.

After 1 month, the patient became septic and was found to have a peri-pancreatic abscess on CT abdomen. IR performed a percutaneous drainage of the peripancreatic abscess with minimal drainage through the drain, without any clinical improvement. EUS was then performed by one of the authors (JN) that revealed a 55 mm, oval, heterogeneous peri-pancreatic fluid collection which had hyperechoic material consistent with the solid debris. A 10mm x 10mm LAMS was then placed from the stomach into the fluid collection with subsequent drainage of pus and debris. The patient clinically improved and was discharged home with improvement of the fluid collection. A follow-up CT Abdomen 4 weeks later showed interval decrease in the size of the previous small fluid collection. The LAMS was uneventfully removed endoscopically at 8 weeks after the initial placement.

## 3. Discussion

Current treatment options for postoperative fluid collections include percutaneous drainage, EUS–guided drainage, and open surgical drainage. Of these, PCD is the current standard of care. Despite its success rate of more than 80%, PCD has numerous disadvantages. Although good results can be achieved with surgery, it is not preferred these days due to relatively higher morbidity and costs [8]. Increasing evidence is accumulating in the literature suggesting EUS-GD as an attractive first-line option due to limitations of PCD.

Limitations of PCD include higher costs compared to EUS-GD due to need for regular monitoring by health care providers, tendency to get obstructed, repeated need for use of disposable health supplies, increased radiation from multiple CT scans, and longer median hospital stay [9–11]. Significant fluid and electrolyte losses, risk of infection, increased morbidity and patient discomfort with an external drain, need for frequent repositioning and drain exchanges, need for repeated drain flushing to maintain patency, and increased risk of long term cutaneous fistula and thereby leading to worse quality of life for patients are some of other disadvantages of PCD [9, 10, 12]. In addition, not all collections are accessible through PCD and PCD could be less successful in infected collections.

### 3.1. Use of Stents for Drainage of PSFC (Table 1)

Currently, there are a large number of studies supporting the use of stents in the treatment of pancreatic pseudocysts and WON [3, 4, 13–15]. Most of the earlier studies involving PSFC treatment have used DPS [6, 7]. More recently, LAMS have been used for a variety of indications including biliary drainage, gallbladder drainage, gastrojejunostomy in gastric outlet obstruction (GOO), EUS–directed transgastric ERCP, drainage of liver abscess, mediastinal abscess, and malignant fluid collections [16–19]. LAMS address the need for larger diameter lumens which can provide direct endoscopic access into a cyst cavity and provide adequate drainage. Specifically, it is becoming more preferred by endoscopists for cystogastrostomy and pancreatic necrosectomy procedures [4, 5].

There are multiple studies in the literature supporting successful use of LAMS for pancreatic fluid collections drainage, but the only study that is available at this time for the use of LAMS in PSFCs of all etiologies is a US multicentre, retrospective analysis of 47 patients by Mudireddy et al. [5]. The authors concluded that the use of LAMS for PSFCs is an attractive alternative option to surgery or PCD for collections situated close to stomach, duodenum, and rectum. The characteristics of collections in this series were inflammation (n=24), abscesses (n=19), and bilomas (n=4). This is similar to our patients who had perigastric abscesses and excellent resolution of abscess cavities was noted. In this study, the clinical success rates were different depending on the site of LAMS placement with highest success rates with transgastric (91.2%), followed by transrectal (87.5%) and then transduodenal (80%) [5]. Notably, the technical and clinical success rates in postpancreatic surgery collections were higher at 100% and 96%, respectively. Another subgroup with high technical and clinical success rates in this study was pelvic fluid collections, which could be attributed to large inner diameter of LAMS allowing better drainage without catheter placement, less chance for stent obstruction by fecal matter, and ability to do debridement as needed [5].

LAMS has many potential advantages compared to plastic stents in PSFC drainage including obviating the need for fluoroscopic guidance as the stent is well visualized under EUS. The possibility of performing the entire procedure under EUS guidance alone is a major advance in therapeutic endoscopy. LAMS are also technically easier to deploy and extract thereby reducing the procedure time and has higher technical success rates [20, 21]. There is reduced risk of leakage with LAMS compared to plastic stents. Due to dual flange anchors and fully covered nature of LAMS, close apposition between the collection and GI lumen can be created minimizing the risk of leakage of secretions into the peritoneum. Saddle shaped design of LAMS makes it less prone to stent migration. LAMS may be more useful in the presence of necrosis due to their wider lumen especially with newer 20 mm diameter stent and providing the endoscopist with direct access for necrosectomy [4, 11]. Superior success rate of LAMS for transrectal drainage of PSFCs was found in one study [7]. Due to the frequent coexistence of solid necrotic component inside the cavity in transrectal fluid collections, these authors did not achieve satisfactory results with the use of DPS and switched to LAMS deployment after the first six cases that were included in their study.

The drawbacks of LAMS include paucity of experience in the use of these stents but that is changing quickly with the wider acceptance of this stent. Higher costs of LAMS at the time of placement is a deterrent compared to PCD [10]. Secondly, due to their wider lumens, LAMS may be more prone to occlusion by necrotic debris and food, thereby increasing the risk of secondary infection. A possible solution to this situation is placement of multiple pigtail stents simultaneously within the lumen of metal cystogastrostomy stents thereby making it difficult for solid particles to pass while continuing to allow for drainage of liquid secretions around the pigtail stents [11, 17]. In addition, LAMS were also found to be associated with higher bleeding rate, including late bleeding at 3-5 weeks in the treatment of postpancreatitis pancreatic fluid collections [22]. EUS guidance with the use of Color Doppler may reduce the risk of intraprocedural bleeding but would not affect delayed bleeding [7]. One hypothesis is that LAMS remained in place even after collapse of the collection without any movement and this causes friction to surrounding vasculature around the necrotic cavity causing increased propensity to bleed [22]. This prompted these authors to change their practice of repeating imaging at 3 weeks to assess the cavity resolution instead of 6 weeks, followed by stent removal if the fluid collection is resolved. Another explanation for increased bleeding events with the use of LAMS is the easier access of low pH fluid with gastric acid into the cyst cavity due to wider lumen of LAMS, thereby causing irritation of exposed intracavitary vessels and increased tendency for bleeding [23]. Notably, no bleeding events were seen in the only study available in the literature on the use of LAMS in PSFCs [5].

Stent migration (both spontaneous and during direct necrosectomy) has also been noted with LAMS possibly due to rapid decompression of cysts with LAMS [14]. Maldeployment of stent was also noted by Siddiqui et al. [4]. In another study of LAMS use in pancreatic collections, pneumoperitoneum and perforation was also reported [5]. Another risk of LAMS is buried stent syndrome usually seen at 3-4 months after placement. LAMS are fairly short (10mm) and immobile. After resolution of PSFCs, due to its lumen apposing nature, the stent may get deeply embedded in the gastric wall with mucosal overgrowth and its removal may be technically challenging requiring an additional endoscopic procedure with sedation [22].

Finally, authors acknowledge that although the procedure was technically successful in all the cases, the final outcome of the patients did not improve in 2 out of the 3 cases, primarily due to the underlying diagnosis of malignancy that adversely affected their prognosis. Therefore, appropriate patient selection is very important in deciding who would benefit from the procedure.

### 3.2. Conclusions and Future Directions

In conclusion, EUS-GD using LAMS is an equally effective but safer and cost-efficient alternative to PCD for treatment of PSFCs. With the increased availability of forward viewing echoendoscopes, the therapeutic possibilities have increased including accessing perisplenic collections and collections around modified anatomy such as Roux-en–Y gastric bypass. With availability of 20 mm diameter LAMS, the efficacy of drainage of PSFCs may even improve further. Further studies for PSFC treatment with EUS-GD using LAMS are warranted for understanding their full scope of use and possibly becoming an established standard of care.

## Figures and Tables

**Table 1:** 

***Authors, Year, Place***	**Number of patients, Study design**	**Indication**	**Stent type and route, size**	**Average size of collection Success rate** **(TS, CS)**	**Time to resolution (TRL) and stent removal (TRO)**	**Average follow up**	**Complication rate**	**Complications**
Mudireddy et al. 2018 US	Multi center, retro spective, n=47	Pancreatic surgery Extra pancreatic surgery Previous failed PCD or surgical drainage (13/47)	LAMS Hot 76% Cold 24% TG 72% TR 17% TD 11% 15mm 70% 10 mm 30%	78.6 mm TS-93.6% (overall) TS- 100% (pancreatic) CS-89.3% (overall) CS- 96% (pancreatic) Necrosectomy 6	TRL- 27.4 d TRO- 36 d	1-8 weeks	Intra-procedural 4.25% Post-procedural 6.4%	Intra-procedural Stent mis deployment N=2, Post procedural Perforation, N=1 Stent migration N=1 Infection N=1

Donatelli et al 2017	Single center, Retrospective, N=32	Post pancreatic N=10 (31%)	DPS	TS 100% CS 93.4%		13.5 months	Immediate 12.5 %	Bleeding N=1 Stent migration N=3(all rectal)

Tellez-Avilla 2015	Retrospective N=43	Pancreatic 4/13 (30.8%) Prior PCD 2/13	DPS Mean size 6.5 cm TG 87.5% TD 12.5 %	TS 100% CS 100%			None	

Gupta 2012 Europe	Retrospective, N=43	Pancreatic surg 57% Extrapancreatic surgery Previous failed PCD or surgical drainage 13/47	DPS TG 85.7% TD 6%	TS CS 80% -8 weeks CS 90% later follow up		60 weeks	14.6%	Early: Bleeding N=4 Stent migration Late: Colonic fistula N=1

Azeem 2012 Mayo	Retrospective, Total N=48 PCD N= 33 EUS GD N=15	Post pancreatic tail resection	DPS FCSEMS(n=1) TP 12/21 TG 13 /21 TD 3/21 Debridement 2/48	7 cm TS 100% CS 80%			9.6%	Delayed bleeding n=2 Stent migration n=1

Varadarajulu 2009 Alabama	Prospective, case series. N=10	Post-distal pancreatectomy Prior PCD N=1	DPS TG 9 /10 TO 1/10	91.4mm TS 100% CS 90%	TRL 8 wk TRO 8 wk	151		Stent migration n=1

Varadarajulu, 2011 Alabama	Retrospective, N=20	Post-distal pancreatectomy Prior PCD or pancreatic stents 10/20	DPS TG 85% TO 15%	78.5 mm TS 100% CS 100%		199 days	None	

Kwon 2013 MSKCC	Retrospective Total N=23 PCD N= 12 EUS –GD N= 9 including (3 with prev PCD)	Post-distal pancreatectomy Previous PCD 3/23	DPPS TG Necrosectomy 4/12	89 mm TS 100% CS 100%	TRL 57 days			Intra-procedural none Post-procedure Small bowel obstruction N=1 Bleeding gastric ulcer N=1

TS: technical success, CS: clinical success, TRL: time to resolution, TRO: time to removal, PCD: percutaneous drainage, LAMS: lumen-apposing metal stent, TG: transgastric, TD: transduodenal, TR: transrectal, TO: transesophageal, DPS: double pigtail stent, FCSEMS: fully covered self-expanding metal stent, and EUS-GD: endoscopic ultrasound-guided drainage.
